# The Effects of PPAR Stimulation on Cardiac Metabolic Pathways in Barth Syndrome Mice

**DOI:** 10.3389/fphar.2018.00318

**Published:** 2018-04-11

**Authors:** Caitlin Schafer, Vicky Moore, Nupur Dasgupta, Sabzali Javadov, Jeanne F. James, Alexander I. Glukhov, Arnold W. Strauss, Zaza Khuchua

**Affiliations:** ^1^The Heart Institute, Cincinnati Children’s Research Foundation, Cincinnati, OH, United States; ^2^The Division of Human Genetics, Department of Pediatrics, University of Cincinnati College of Medicine and Cincinnati Children’s Research Foundation, Cincinnati, OH, United States; ^3^Department of Physiology, School of Medicine, University of Puerto Rico, San Juan, Puerto Rico; ^4^Medical College of Wisconsin, Milwaukee, WI, United States; ^5^Department of Biochemistry, I.M. Sechenov First Moscow State Medical University, Moscow, Russia; ^6^Faculty of Biology, Lomonosov Moscow State University, Moscow, Russia

**Keywords:** PPAR agonist, bezafibrate, cardiomyopathy, Ostn, musclin, Barth syndrome, tafazzin

## Abstract

**Aim:** Tafazzin knockdown (TazKD) in mice is widely used to create an experimental model of Barth syndrome (BTHS) that exhibits dilated cardiomyopathy and impaired exercise capacity. Peroxisome proliferator-activated receptors (PPARs) are a group of nuclear receptor proteins that play essential roles as transcription factors in the regulation of carbohydrate, lipid, and protein metabolism. We hypothesized that the activation of PPAR signaling with PPAR agonist bezafibrate (BF) may ameliorate impaired cardiac and skeletal muscle function in TazKD mice. This study examined the effects of BF on cardiac function, exercise capacity, and metabolic status in the heart of TazKD mice. Additionally, we elucidated the impact of PPAR activation on molecular pathways in TazKD hearts.

**Methods:** BF (0.05% w/w) was given to TazKD mice with rodent chow. Cardiac function in wild type-, TazKD-, and BF-treated TazKD mice was evaluated by echocardiography. Exercise capacity was evaluated by exercising mice on the treadmill until exhaustion. The impact of BF on metabolic pathways was evaluated by analyzing the total transcriptome of the heart by RNA sequencing.

**Results:** The uptake of BF during a 4-month period at a clinically relevant dose effectively protected the cardiac left ventricular systolic function in TazKD mice. BF alone did not improve the exercise capacity however, in combination with everyday voluntary running on the running wheel BF significantly ameliorated the impaired exercise capacity in TazKD mice. Analysis of cardiac transcriptome revealed that BF upregulated PPAR downstream target genes involved in a wide spectrum of metabolic (energy and protein) pathways as well as chromatin modification and RNA processing. In addition, the Ostn gene, which encodes the metabolic hormone musclin, is highly induced in TazKD myocardium and human failing hearts, likely as a compensatory response to diminished bioenergetic homeostasis in cardiomyocytes.

**Conclusion:** The PPAR agonist BF at a clinically relevant dose has the therapeutic potential to attenuate cardiac dysfunction, and possibly exercise intolerance in BTHS. The role of musclin in the failing heart should be further investigated.

## Introduction

Peroxisome proliferator-activated receptors (PPARs) are members of the nuclear receptor family, a group of ligand-inducible transcription factors involved in the regulation of diverse biological functions, including lipid metabolism (Duval et al., 2007), energy homeostasis, immune response, and inflammation (van Raalte et al., 2004). There are three isoforms of PPAR receptors that have specific, but also overlapping target genes: PPARα, PPARβ/δ, and PPARγ. PPAR subtypes exhibit distinct tissue expression patterns and regulate diverse biological processes. PPARs are activated by subtype-specific or pan-PPAR agonist ligands, such as long-chain fatty acids, or pharmacological activators. Activation of PPARs by respective agonist ligands triggers conformational changes in PPAR molecular structure (Bernardes et al., 2013) and promotes their nuclear translocation and recruitment of nuclear receptor coactivators such as RXR and PGC-1α. A ligand-activated PPAR-coactivator complex regulates the transcription of genes by binding to their peroxisome proliferator response elements (PPREs).

Pharmacological activators of PPARs have been shown to lower plasma and liver triglycerides and improve energy metabolism in several disease models. PPARs are therapeutic targets for correction of inborn errors of fatty acid oxidation (FAO) disorders (Djouadi and Bastin, 2008), dyslipidemia (Duval et al., 2007), and type-2 diabetes (Gross and Staels, 2007). Long-term treatment with pan-PPAR agonist bezafibrate (BF) improved aging-like phenotypes in mice with mutated mitochondrial DNA polymerase c (*Polg*) (Dillon et al., 2012). PPAR agonists showed promising neuroprotective effects in mouse models of tauopathy, mitochondrial encephalopathy, and Huntington’s disease (Dumont et al., 2012; Johri et al., 2012; Noe et al., 2013). Activation of PPARα with its agonist GW7647 prevented post-ischemic contractile dysfunction in neonatal rabbit hearts (Lam et al., 2015) and slowed progression of left ventricular (LV) dysfunction in porcine model of tachycardia-induced cardiomyopathy (Brigadeau et al., 2007). PPARα is implicated in cardioprotective signaling of AMPK through mitochondria (Barreto-Torres et al., 2012; Barreto-Torres and Javadov, 2016). In addition to the ability to stimulate FAO and mitochondrial biogenesis, PPARα can regulate mitochondrial function through interaction with cyclophilin D, a major regulator of the permeability transition pore (Barreto-Torres et al., 2015; Barreto-Torres and Javadov, 2016). Differential transcriptome analysis of the mouse liver revealed that commonly used synthetic PPARα agonist Wy14643 and fenofibrate upregulated sets of genes that are involved in FAO, and downregulated those involved in different aspects of immunity and inflammation (Szalowska et al., 2014). On the other hand, pan-PPAR agonist BF failed to show any beneficial effects in the recent trial on patients with palmitoyltransferase II deficiency, where primary endpoints were exercise-induced changes of FAO velocity and heart rate, which would indicate improved exercise tolerance (Orngreen et al., 2014a). In another study, long-term treatment of mice with BF had no impact on electron transport chain (ETC) activity in the skeletal muscle, but was accompanied by severe metabolic side effects in the liver and hepatomegaly (Yatsuga and Suomalainen, 2012). It is plausible that the activation of PPAR pathways induces distinct effects in various tissues.

Recently, we examined the therapeutic potential of BF in a mouse model of Barth syndrome (BTHS) (Huang et al., 2017). BTHS is a multisystem metabolic genetic disease that is caused by mutations in the tafazzin (Taz) gene on X-chromosome. Taz is a mitochondrial transacylase that is critical for the biogenesis of mitochondrial phospholipid cardiolipin, the signature protein of the mitochondrial inner membrane. Cardiolipin is essential for the maintenance of mitochondrial cristae architecture, structural integrity of ETC complexes, and mitochondrial ATP synthesis. In mice, Taz deficiency presents with diminished exercise capacity and age-dependent dilated cardiomyopathy. Supra-pharmacological dose of BF (600–800 mg/kg body weight) effectively prevented cardiomyopathy and preserved systolic function in Taz knockdown (TazKD) mice, a mouse model of BTHS. However, it is unclear whether BF can attenuate cardiac dysfunction at a lower, clinically relevant dose (10–60 mg/kg body weight). Moreover, molecular targets of BF-mediated PPAR activation in heart are largely unknown. In the present study, we examined the therapeutic potential of BF on cardiac function and exercise capacity in a clinically relevant dose of BF. Additionally, we studied the differential transcriptome as well as the transcriptional impact of PPAR activation in hearts of TazKD mice. We identified molecular biomarkers that were significantly impacted in hearts by Taz deficiency. One of the identified markers, *Ostn*, encodes small myokine musclin that shares C-terminal homology with natriuretic peptides. *Ostn* is mainly expressed in developing osteoblasts and fast glycolytic skeletal muscles (Moffatt et al., 2007; Subbotina et al., 2015). Expression of *Ostn* in normal heart is very low.

## Materials and Methods

### Animals

All animal studies were approved by the Institutional Animal Care and Use Committee of Cincinnati Children’s Hospital Medical Center. Animals were housed in microisolator cages at 25°C under a 14/10 h light/dark cycle with free access to drinking water and food. Doxycycline-inducible shRNA-mediated TazKD mice have been described previously (Soustek et al., 2010; Acehan et al., 2011). Taz knockdown was induced prior to conception by feeding females doxycycline-containing rodent chow (625 mg/kg) 3 days before mating. This approach allows 85–95% silencing of Taz in the heart and skeletal muscle (Acehan et al., 2011). In the ensuing offspring, after 3 months of age, the doxycycline administration route was switched to drinking water (0.05% doxycycline, 10% sugar). Both wild type (WT) and TazKD mice were continuously maintained on doxycycline-containing water for the duration of the study. Male mice with C57BL/6J background were used in experiments.

At 3 months of age, mice were given specifically formulated pelleted rodent chow that contained either no or 0.05% BF (TestDiet, St. Louis, MO, United States) provided *ad libitum* for 4 months. At the same time, the doxycycline administration route was changed for all animals from rodent chow to drinking water with 0.05% of doxycycline and 10% sucrose since manufacturing and sterilizing the rodent chow that contained both BF and doxycycline were technically difficult.

### Exercise Tests

#### Voluntary Exercise on the Running Wheel

A group of mice were placed in individual cages with running wheels that were online with the computer (Columbus Instrument, Columbus, OH, United States). Distance ran by each mouse was recorded daily.

#### Treadmill Exercise

The exercise test was performed on a 6-line motorized treadmill with adjustable speed, inclination, and equipped with an electric shock-delivering grid (Columbus Instruments, Columbus, OH, United States). All mice were pre-acclimatized to experimental settings the day prior by running for 15 min at a speed of 10 m/min with disabled electric shock-delivering grid. During the test, the treadmill program consisted of 5 min of warm-up at 5 m/min and then 25 m/min. Electric shock intensity was set to 1 mA. Each mouse was allowed three falls, which were determined when the mouse would rather receive the shock than run. The hind limbs or body resting on the shock pad for 3 s was counted as a “fall.” The total distance each mouse ran was recorded. Each mouse was subjected to tests in two separate sessions, 10 days apart.

### Echocardiography

Two-dimensional and M-mode transthoracic echocardiography were performed under isoflurane anesthesia (1.75% isoflurane) as previously described using a Vevo 2100 Micro-Imaging system (VisualSonics, Inc.) and a 40 MHz transducer (Acehan et al., 2011; Huang et al., 2017). Off-line analyses were performed by investigators blinded to treatment and genotype.

### RNA Extraction, Library Preparation, and RNA Sequencing

Left ventricles from 7-month old mice were processed for RNA extraction using the TRIzol Reagent according to the manufacturer’s protocol. Human cardiac LV tissues were obtained from CCHMC biorepository.

RNA was quantified in Bioanalyzer 2100. RNA was frozen and stored in -80°C immediately after production. RNA quality was assessed by running the samples on Agilent RNA 6000 Nano-gels. For each total RNA library (5 μg) ribosomal RNA was removed using Invitrogen’s RiboMinus Kit (No. A10837-08) and then samples were concentrated using the RiboMinus Concentration Module (Invitrogen).

Sequencing libraries were generated using the NEBNext^®^ Ultra^TM^ RNA Library Prep Kit for Illumina^®^ (NEB, United States) according to the manufacturer’s instructions. The Agilent Bioanalyzer 2100 system was used to evaluate the quality of libraries. Library preparations were sequenced on the Illumina HiSeq platform, with 40 million reads. RNA-sequencing results were deposited at the Sequence Read Archive at NCBI (Accession ID SRP132110). For bioinformatics analysis, BAM files were submitted to AltAnalyze software package (v.2.1.0).

### Bioinformatics

RNA-seq reads were aligned to the mouse reference sequence (GRCm38) using TopHat, and transcripts were assembled with Cufflinks package (Trapnell et al., 2012). Differential expression analysis, pathway enrichment, and disease network visualization were performed using AltAnalyze software v.2.1.0 with integrated Cytoscape package (Soreq et al., 2015). Additional gene enrichment analysis was done using ToppGene Suite (Chen et al., 2009).

### Quantitative RT-PCR

Total RNA was quantified with NanoDrop^TM^ (NanoDrop, Thermo Fisher Scientific, United States). Each sample (50 ng) was submitted to reverse transcriptase using the High-Capacity cDNA Reverse Transcription Kit (Applied Biosystems, United States), according to manufacturer’s instructions. Osteocrin (*OSTN*) transcript quantitative analysis was performed using commercial TaqMan^®^ probe-based assay (Thermo Fisher Scientific, United States), specific to human (assay ID Hs00893469_m1) and mouse (assay ID Mm00813799-m1) species. Glyceraldehyde-3-phosphate dehydrogenase (*GAPDH*) was selected as a housekeeping control gene (assay IDs Hs03929097-g1 and Mm99999915-g1 for human and mouse species, respectively). Analysis was performed on the Realplex Mastercycler system (Eppendorf). Relative expression values of each gene were calculated applying the ΔΔCt fold change (FC) method.

### Protein Analysis by Western Blotting

Western immunoblot analysis was performed using Novex^TM^ 10–20% Tricine protein gels (Thermo Fisher Scientific, United States). Blood was collected from the femoral veins of 3 month-old WT and TazKD mice and was allowed to clot. The clot was removed by centrifugation at 3000 × *g* for 15 min at room temperature. Serum was diluted with SDS–PAGE loading buffer and 25 μl of samples was loaded on each well. Electrophoresis was performed for 90 min at 125 V. Proteins were transferred to nitrocellulose membranes using the semi-dry transfer method. Membranes were blocked overnight at 4°C with agitation using TBS (10 mM Tris, 150 mM NaCl) supplemented with 5% BSA. Membranes were then washed 3 × 5 min with 0.02% Tween-20 in TBS and incubated overnight at +4°C with primary rabbit polyclonal antibodies specific to mouse muslin (CAU21957, Biomatik, Wilmington, DE, United states) with agitation. Membranes were washed 3× with 0.02% Tween-20 in TBS. Secondary goat anti-rabbit IRDye antibodies were used for imaging (Licor Biosciences, Lincoln, NE, United States). The secondary antibodies were used at a concentration of 1:20,000, with an incubation period of 1 h at room temperature. The Odyssey CLx scanner was used for imaging of membranes.

### Statistical Analysis

Data are presented as means ± standard deviation. Statistical significance was evaluated using one-way ANOVA followed by Tukey’s multiple comparison *post hoc* test, or an unpaired two-tailed Student’s *t*-test. Differences were considered to be statistically significant when *P* < 0.05.

## Results

### Long-Term Uptake of 0.05% Bezafibrate Ameliorates Cardiomyopathy in TazKD Mice

**FIGURE 1:**
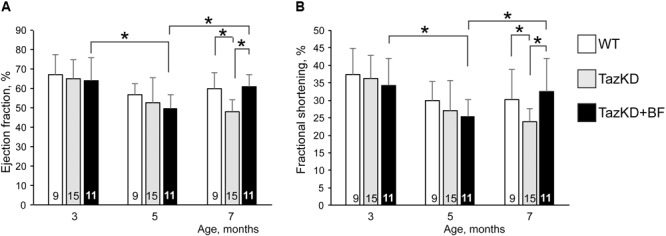
Bezafibrate (BF) ameliorates systolic function in TazKD mice. **(A)** Left ventricular ejection fraction. **(B)** Left ventricular fractional shortening. Asterisks denote significant differences between groups (^∗^*p* < 0.05).

In the first set of experiments, we investigated whether the BF at clinically relevant doses might ameliorate cardiomyopathy and improve systolic function in TazKD mice. An experimental group of 2.5 months old TazKD mice were given rodent chow that contained 0.05% of BF. The control group received a standard rodent lab diet. Cardiac function was examined by echocardiography at 3, 5, and 7 months of age in untreated WT, untreated TazKD, and BF-treated TazKD groups. Analysis of EF and FS dynamics revealed a significant reduction of LV ejection fraction (EF) and fractional shortening (FS) between 3 and 5 months of age both in untreated and BF-treated TazKD groups. However, EF and FS indices were not statistically different from age-matched untreated WT controls. As expected, untreated TazKD mice developed significant systolic impairments by 7 months of age, which is evident by reduced EF and FS, compared to the age-matched untreated WT group (**Figures 1A,B** and Supplementary Table S1).

Intake of BF with food during the 4-month period effectively prevented the development of systolic dysfunction in 7 months old TazKD mice. Interestingly, the EF and FS indices showed worsening dynamics from 3 to 5 months of age both in untreated and BF-treated TazKD groups. However, this trend was reversed and systolic function improved between 5 and 7 months of age in the BF-treated TazKD mice, while in the untreated TazKD group it continued to further deteriorate.

### Bezafibrate in Combination With Voluntary Exercise Improves Exercise Capacity in TazKD Mice

**FIGURE 2:**
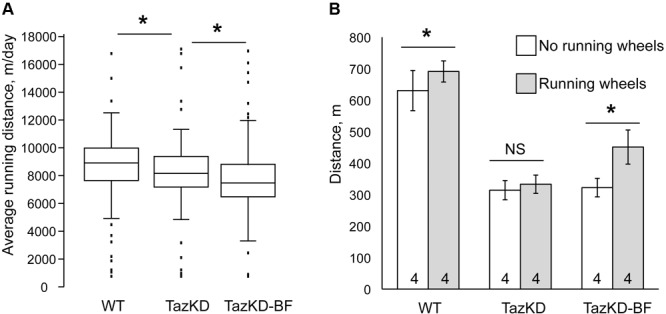
Exercise performance of mice on the treadmill. **(A)** Distance traveled on the treadmill until exhaustion of WT-, TazKD-, and BF-treated TazKD mice (TazKD-BF) that were housed with or without in-cage running wheels. Asterisks denote significant differences between groups (^∗^*p* < 0.05). **(B)** Average daily running distance of WT-, untreated-, and BF-treated TazKD mice on running wheels. Asterisks denote significant differences between groups (*p* < 0.05).

TazKD mice have significantly impaired exercise capacity that is consistent with the exercise intolerance phenotype in human patients with BTHS (Acehan et al., 2011; Clarke et al., 2013; Powers et al., 2013). When subjected to involuntary exercise test on the treadmill, TazKD mice repeatedly failed to stay on the treadmill belt and in average ran approximately 35% less distance than WT controls. Treatment with BF alone had no notable effect on the exercise capacity of TazKD mice (**Figure 2B**). We investigated whether a voluntary exercise on the running wheel had an effect on the exercise capacity in BF-treated TazKD mice. To this end, a group of 3 months old WT and TazKD mice were housed in cages that were equipped with running wheels for 4 months. Untreated TazKD mice ran in average 9% less distance than WT controls (*P* < 0.0001) on running wheels. BF-treated TazKD mice ran in average 6% less distance than untreated TazKD mice (*P* < 0.02) and 16% less distance than WT controls (*P* < 0.0001) on running wheels.

After 4 months of housing in cages with running wheels, the mice were subjected to a treadmill test. Voluntary exercise on the running wheel significantly potentiated the effect of BF in subsequent running capacity on the treadmill in TazKD mice (**Figures 2A,B**). TazKD mice that had access to running wheels performed in average 40% better than TazKD mice without running wheels (*P* < 0.0001). In the treadmill tests no significant differences were found between performances of untreated TazKD mice with and without running wheels. WT mice with access to running wheels performed slightly better (9.6%, *P* < 0.03) on the treadmill than those without wheels.

### Bezafibrate Treatment Exerts Divergent Effects on Metabolic Genes in TazKD Hearts

In order to identify genes altered by pharmacological PPAR activation in myocardium, we performed RNA-seq analysis of hearts from WT-, TazKD-, and BF-treated TazKD mice. Principal component analysis and counts of transcript features are presented in the Supplementary Figure S1. Taz deficiency caused downregulation of 52 genes and upregulation of 188 genes with *P*-values≤0.05 (FDR) and FC cut-off ≥2.0. In BF-treated TazKD hearts, 3161 genes were significantly upregulated (Supplementary Table S4). However, statistical analysis failed to reveal any significantly downregulated genes in the BF-treated group, which met the cut-off criteria.

To overview the biological processes in TazKD heart affected by long-term uptake of BF, significantly regulated genes in untreated and BF-treated TazKD hearts were subjected to Gene Ontology (GO) analysis using AltAnalyze and ToppGene software (Chen et al., 2009; Soreq et al., 2015).

**FIGURE 3:**
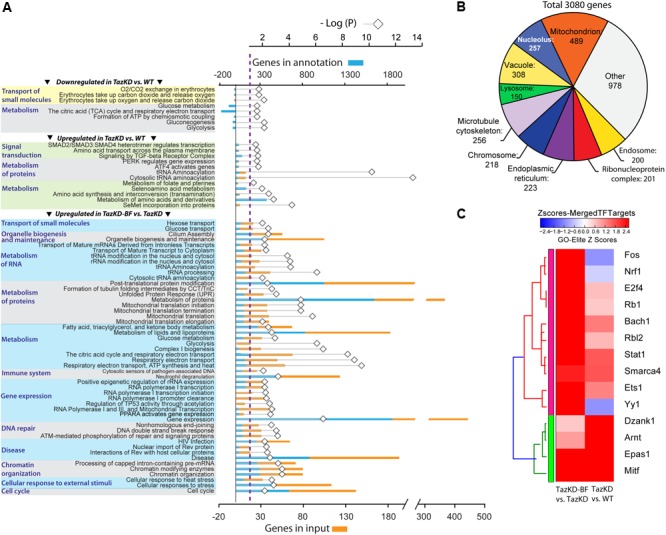
Pathways that are affected by BF treatment in TazKD hearts. **(A)** Gene Ontology (GO) terms (FDR < 0.05) significantly enriched in regulated genes in hearts of TazKD- and BF-treated TazKD mice. **(B)** Subcellular localization of proteins, encoded by upregulated genes in BF-treated TazKD mice. **(C)** The heatmap of differentially expressed transcription factors in BF-treated TazKD hearts.

The GO analyses performed for downregulated genes in untreated TazKD hearts showed that GO terms related to energy metabolism (glycolysis, gluconeogenesis, TCA cycle, and ETC) were significantly enriched. Also, genes involved O_2_/CO_2_ exchange processes were downregulated in untreated TazKD hearts. Significantly upregulated genes were those involved in metabolism of proteins, folate, amino acids, and tRNAs. Additionally, upregulated genes in TazKD hearts were enriched with GO terms related to signal transduction processes (ATF4, SMAD2/SMAD3/SMAD4, TGF-beta, and PERK) (**Figure 3A**).

Prolonged BF uptake resulted in robust activation of genes involved in a wide-spectrum of processes. Upregulated genes in BF-treated TazKD hearts were enriched with GO terms related to energy metabolism (metabolism of fatty acid, ketone bodies, and glucose), metabolism of proteins, mitochondrial protein transport, RNA metabolism, gene expression, DNA repair, chromatin organization, immune system, and organelle biogenesis and maintenance (**Figure 3A**). The majority of significantly upregulated genes encode mitochondrial proteins (**Figure 3B**). More detailed pathway diagrams with highlighted regulated genes are presented in Supplementary Figures S2–S11.

Differentially expressed genes datasets were enriched with target genes of several important transcription factors (**Figure 3C**). Targets genes of Nrf1, Fos, and Yy1 were downregulated in untreated TazKD group and upregulated in BF-treated TazKD group (**Figure 3C**).

**FIGURE 4:**
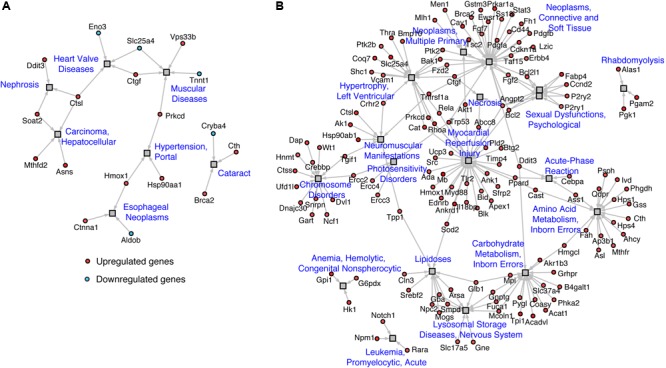
Disease network of regulated genes identified in **(A)** WT vs. TazKD and **(B)** TazKD vs. TazKD-BF datasets. Rectangles and circles correspond to disorders and disease genes, respectively. A link is placed between a disorder and a disease gene if mutations in that gene lead to a specific disorder. Network is generated with AltAnalyze software interrogating the Comparative Toxicogenomics Database (http://ctdbase.org/).

Mapping of regulated genes from our datasets on the human disease network revealed that 25 genes in TazKD hearts were associated with muscular disease, heart valve disease, nephrosis, cataract, hepatocellular carcinoma, and esophageal neoplasms (**Figure 4A**). Additionally, the 189 genes that are changed in BF-treated hearts were associated with various human pathological conditions, such as LV hypertrophy, myocardial reperfusion injury inborn errors in carbohydrate and amino acid metabolism, lysosomal disease, and others (**Figure 4B**).

### Secreted Myokine Musclin Is Robustly Produced in TazKD Hearts

**FIGURE 5:**
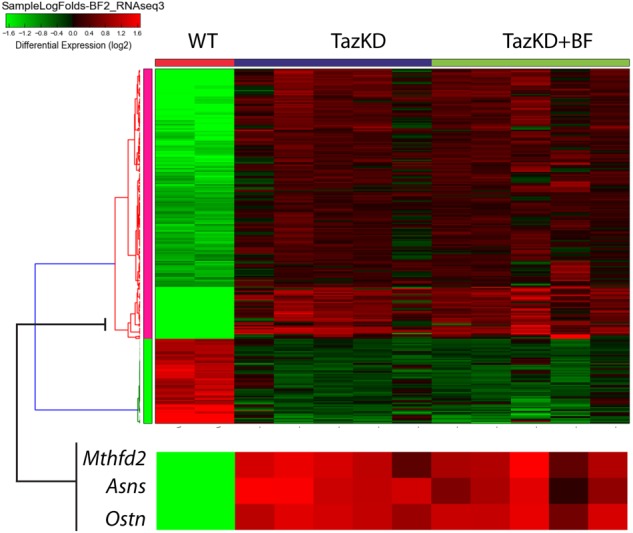
The clustered heatmap of differentially expressed genes in rows of untreated WT, untreated TazKD, and BF-treated TazKD groups. Log2 values of FCs of 262 differentially expressed genes are shown. Expression values intensities in rows are normalized to row means. Rows were clustered using the average Euclidian method. Zoomed fragment of the heatmap with the expression FC values of *Asns, Ostn, Mthfd2*, and *Atf4* are magnified on the lower pane.

**FIGURE 6:**
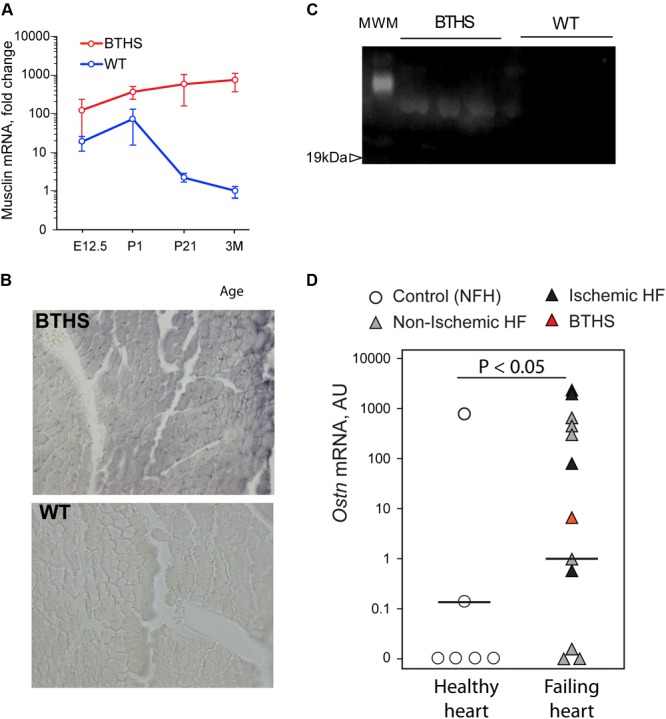
Musclin is upregulated in failing hearts. **(A)** Musclin mRNA in developing and adult heart (*Y*-axis is in the logarithmic scale). **(B)** DAB-staining of 3 months old left ventricles with anti-musclin antibodies. **(C)** Western blot of circulating musclin in plasma of TazKD mice. **(D)** Quantification of *Ostn* mRNA in non-failing and failing human hearts with Taqman assay.

Analysis of our RNA-seq data on transcript level revealed potent upregulation of *Mthfd2, Asns*, and *Ostn* (**Figure 5**). We found remarkable upregulation of the *Ostn* gene in TazKD hearts, while expression of *Ostn* in WT hearts was very low. To further investigate the role of *Ostn* induction in the heart we analyzed the expression of *Ostn* in normal and TazKD hearts at different developmental stages (**Figure 6A**). *Ostn* is expressed in embryonic hearts both in WT and TazKD mice. After birth (P1), *Ostn* expression in WT hearts sharply declines, and by the weaning age (P21) the *Ostn* mRNA in the heart is barely detectable. In contrast, in TazKD hearts *Ostn* expression is retained in the adulthood. Treatment with BF caused further upregulation of *Ostn* in TazKD hearts (Supplementary Table S4).

Staining of LV sections of adult (3-month-old) WT and TazKD mouse hearts with musclin-specific antibodies demonstrated that musclin protein was present in TazKD hearts, and was virtually absent in WT cardiac tissues (**Figure 6B**). With immunoblot analysis, we detected circulating musclin in the plasma of adult TazKD mice (**Figure 6C**). These results clearly demonstrate that musclin is not produced in the adult mouse myocardium, but is robustly induced in TazKD heart.

Next, we investigated whether musclin is induced in failing human hearts. Tissue samples of 12 failing hearts, including one explant from a 1-year-old patient with BTHS and six non-failing controls were obtained from CCHMC biorepository. RNA was isolated from frozen heart samples and *Ostn* mRNA levels were determined with the Taqman quantitative PCR assay. Analysis demonstrated that 9 out of 12 failing hearts (including the one with BTHS) expressed high level of *Ostn*, while 4 out of 6 healthy hearts had no detectable levels of *Ostn* mRNA (**Figure 6D**). There was no clear difference in *Ostn* expression levels between ischemic and non-ischemic failing hearts.

## Discussion

Peroxisome proliferator-activated receptor signaling promotes a transcriptional activation of FAO and mitochondrial metabolism genes in the liver and adipose tissues. BF is a pan-PPAR activator and is widely used for the treatment of hypertriglyceridemia and hypercholesterolemia. There are major discrepancies in BF studies in humans and rodents. In clinical practice, BF is typically prescribed at daily dosage 10–25 mg/kg (Yamaguchi et al., 2012; Orngreen et al., 2014a,b). However, in rodent studies BF is commonly introduced with rodent food, where the drug content is 0.5% w/w that corresponds to a daily dose of 600–800 mg/kg (Dillon et al., 2012; Dumont et al., 2012; Huang et al., 2017). It is unclear whether the supra-pharmacological doses of BF used in rodents are comparable to human studies. Detailed study on PPARα knockout mice suggested that low-dose BF in the clinically relevant dose decreases serum and liver triglycerides in a PPARα-independent manner by down-regulating sterol regulatory element-binding protein 1c (*Srebp-1c*) and attenuating hepatic lipogenesis and triglyceride secretion (Nakajima et al., 2009). The majority of published studies are focused on the lipid-lowering actions of BF with an emphasis on liver, adipose tissues, or skeletal muscles, whereas relatively few have investigated protective actions of BF in the heart (Tenenbaum et al., 2005; Brigadeau et al., 2007; Bulhak et al., 2009; Huang et al., 2017). It is unclear whether BF in clinically relevant doses has any effect on energy metabolism in cardiomyocytes in rodents.

The present study, to our knowledge, is the first report demonstrating cardioprotective effects of BF at a dose of 0.05%. This is 10 times lower than the dose used in previous studies and 2.4–8 times higher than the commonly prescribed dose for treatment of patients with dyslipidemia. We showed that during the first 8 weeks of BF intake, between 3 and 5 months of age, the cardiac function continuously deteriorates. However, systolic function is markedly improved between 8 and 16 weeks of BF intake, suggesting that long-term intake of BF is necessary for the improvement LV function.

Interestingly, while BF prevented the development of systolic dysfunction and cardiomyopathy in the mouse model of BTHS, it failed to ameliorate impaired exercise capacity, another BTHS phenotype that is also mimicked in TazKD mice. This finding is consistent with the results of a recent trial, where BF failed to improve FAO rates in skeletal muscles of patients with carnitine palmitoyltransferase II and very long-chain acyl-CoA dehydrogenase deficiencies (Orngreen et al., 2014b). Similarly, in another study 0.5% BF failed to induce mitochondrial biogenesis in skeletal muscles of two mouse genetic models with skeletal myopathies (Yatsuga and Suomalainen, 2012). Our results show that BF induces more strong effects in heart than in skeletal muscles. It is also conceivable that BF may evoke distinct responses in various types of muscle fibers, which contain different PPAR subtypes. In skeletal muscles, PPARβ/δ is several fold more abundant than either PPARα or PPARγ (Muoio et al., 2002). In the skeletal muscle, PPARβ/δ controls the expression of PGC1α, whereas the effects of BF are mediated in large part by PPARα (Peters et al., 2003; Schuler et al., 2006).

When combined with voluntary exercise on the running wheel, BF markedly improved exercise capacity in TazKD mice in the subsequent treadmill test. Underlying mechanisms of the synergistic effect of BF with everyday voluntary exercise are unclear. It is plausible that simultaneous exercise-induced activation of master regulators of energy metabolism and/or epigenetic modifications is involved. Physical activity elicits complex responses on signaling pathways in skeletal muscles that are coupled with downstream regulators of transcription and translation. Molecular effectors of physical exercise include AMPK, ATF2, CREB, FOXOs, NRFs, CaMKs, MEF2, p38/MAPK, PGC family, and other master regulators of metabolism [reviewed in Egan and Zierath (2013)]. Concomitant activation of these signaling systems along with PPAR signaling may potentiate BF effects on skeletal muscles. Additionally, exercise may significantly alter the cellular epigenetic landscape, facilitate transcriptional activation of the PPAR-target genes, and enhance metabolic plasticity of skeletal muscles. It has been reported that HDAC4 and 5 were excluded from the nucleus in response to acute exercise, thereby removing their transcriptional repressive function (McGee et al., 2009). Exercise reduces association of HDAC5 with MEF2 and promotes association of MEF2 with PGC-1α (McGee, 2007).

Taken together, published reports and our results strongly suggest that BF is a promising therapeutic agent that shows cardioprotective potential in various models of cardiac injury. However, the therapeutic effects of BF on skeletal muscles should be evaluated in more detail on molecular level.

In our study, we identified 52 downregulated and 188 upregulated genes in untreated TazKD mice compared to WT controls (Supplementary Tables S2, S3), and over 3000 up-regulated genes in the hearts of TazKD mice after 4-month treatment with the BF (Supplementary Table S4). GO analysis revealed genes that are involved in O_2_/CO_2_ exchange processes were downregulated in TazKD hearts compared to WTs. This observation is consistent with clinical evidence: patients with BTHS have markedly reduced ability to extract oxygen from blood, even though the blood is oversaturated with oxyhemoglobin (Spencer et al., 2011). Genes involved in glucose metabolism, TCA cycle, and ATP generation were also significantly downregulated in TazKD hearts. Treatment with BF resulted in upregulation of genes that are involved in energy production, metabolism of proteins and amino acids, gene expression, processes of RNA modifications, and transport. Upregulation of energy metabolism pathways (FAO, glycolysis, TCA cycle, and ETC) suggests that PPAR activation with BF improves bioenergetics in TazKD heart. GO analysis revealed the upregulation of genes involved in chromatin organization, suggesting that PPAR activation may evoke epigenetic changes in cardiomyocytes.

We identified a set of genes that were downregulated in untreated TazKD hearts and upregulated in BF-treated TazKD hearts. Interrogation of ENCODE database revealed that these genes are targets of key transcription factors Nrf1, Fos, and Yy1. Nuclear respiratory factor 1 (NRF1) activates the expression of a wide range of nuclear genes that are essential for mitochondrial biogenesis, regulation of genes which encode mitochondrial respiratory complex subunits, heme biosynthetic enzymes, and regulatory factors involved in the replication and transcription of mitochondrial DNA. Furthermore, NRF1 target genes play a pivotal role in the regulation of extra-mitochondrial biological processes, including RNA metabolism, splicing, cell cycle, DNA damage repair, protein translation initiation, and ubiquitin-mediated protein degradation (Satoh et al., 2013). FOS regulates TGF-beta signaling, which plays an important role in the pathogenesis of cardiac fibrotic and hypertrophic remodeling (Dobaczewski et al., 2011). YY1 is a negative regulator of α-myosin heavy chain promoter and plays an important role in cardiac differentiation (Sucharov et al., 2003; Gregoire et al., 2013). We did not find significantly downregulated genes in BF-treated group. While surprising, this finding is consistent with the transcriptional signature of another PPAR agonist Wy14643 in hepatocytes (Szalowska et al., 2014).

Gene-level analysis revealed that TazKD hearts highly express *Mthfd2* that encodes a rate-limiting enzyme in mitochondrial tetrahydrofolate metabolism. Increased abundance of MTHFD2 in TazKD cardiac mitochondria has been previously demonstrated in the proteomics analysis (Huang et al., 2015). It is conceivable that upregulation of folate metabolism is an adaptive response which enhances the mitochondrial innate antioxidant defense system in BTHS hearts. A recent study has shown that the major source of NADPH in mitochondria is MTHFD2 (Fan et al., 2014), and NADPH plays a central role in mitochondrial ROS scavenging (Nickel et al., 2015).

*Asns*, another highly upregulated gene in TazKD hearts, encodes the asparagine synthetase, which serves as a nutritional regulator in the cell and is essential for cellular adaptation to stress (Zhang et al., 2014; Toda et al., 2016). *Asns* is transcriptionally modulated by ATF4, a member of the family of cAMP-response element-binding proteins (CREBs), which is concomitantly upregulated in TazKD hearts.

RNAseq and subsequent immunochemical analysis revealed robust expression of *Ostn* in TazKD hearts, whereas in WT hearts *Ostn* mRNA is barely detectable. We found that *Ostn* is also markedly upregulated in failing human hearts. *Ostn* encodes a small metabolic hormone musclin, which is produced by developing osteoblasts and fast glycolytic skeletal muscles where its expression is stimulated by physical exercise (Subbotina et al., 2015). Recently, it has been discovered that *Ostn* is expressed in primate neocortex and restricts activity-dependent dendritic growth in human neurons (Ataman et al., 2016). Musclin has a homology with natriuretic peptides (NPs) and binds with natriuretic clearance receptor (NPR3) with high affinity and promotes mitochondrial biogenesis. The biological effect of musclin is mediated through increased circulating levels of NP A, which binds to NPR1 and activates GPCR and PGC-1α pathways (Moffatt et al., 2007; Subbotina et al., 2015; Chiba et al., 2016). Recent study showed that musclin exhibits cardioprotective role and reduces acute inflammation after myocardial infarction in mice (Miyazaki et al., 2018). Musclin might affect local concentrations of NPs and augment cardiac function by decreasing the preload and exerting anti-inflammatory effects in failing heart (Kiemer and Vollmar, 2001). Musclin could be insensitive to plasma membrane-bound neutral endopeptidase (NEP) that breaks the Cys–Cys bond for the ring structure of NPs. Because both musclin and NPs bind to NPR3, the former can inhibit the clearance of NPs and enhance NP-dependent signaling in NPR3-expressing tissues (Miyazaki et al., 2018).

In summary, our study allows to draw several important conclusions: (a) The heart is highly responsive to the treatment with pan-PPAR agonist, BF, which at a clinically relevant dose effectively prevents the development of cardiomyopathy in the mouse model of BTHS. (b) The skeletal muscle is less responsive to BF treatment, however, voluntary exercise potentiates the BF action in TazKD mice. (c) BF-mediated activation of PPAR signaling upregulates a wide spectrum of pathways related to energy metabolism, protein metabolism, gene expression, chromatin modification, and RNA processing. (c) Taz knockdown in hearts induces downregulation of genes related to O_2_/CO_2_ exchange and aerobic metabolism, and upregulation of pathways of amino acid metabolism, tRNA metabolism, folate metabolism, and several cellular signaling systems, with involvement of PERK, TGF-beta, ATF4, and SMADs. (d) The metabolic hormone musclin, which is not synthesized in normal myocardium, is highly expressed in failing hearts, plausibly inducing compensatory mitochondrial biogenesis in energy-deprived cardiomyocytes. The elucidation of molecular targets of PPAR agonists and metabolic pathways that are governed by these target genes may lead to the discovery of new pharmacological agents and the repurposing of existing drugs for therapeutic intervention in cardiac diseases.

## Ethics Statement

This study was carried out in accordance with the recommendations of the Institutional Animal Care and Use Committee of Cincinnati Children’s Hospital Medical Center. The protocol was approved by the Institutional Animal Care and Use Committee of Cincinnati Children’s Hospital Medical Center.

## Author Contributions

ZK designed the studies. CS and ZK performed the experiments. VM and JJ performed the echocardiography and analyzed the data. ND performed the bioinformatic analysis. SJ, AS, AG, and ZK contributed to the interpretation of data and critically revised the paper. All authors reviewed and approved the final version of the paper.

## Conflict of Interest Statement

The authors declare that the research was conducted in the absence of any commercial or financial relationships that could be construed as a potential conflict of interest.
