# Supplementary material for: The Effects of PPAR Stimulation on Cardiac Metabolic Pathways in Barth Syndrome Mice
Source: Front Pharmacol. 2018 Apr 11;9:318. doi: 10.3389/fphar.2018.00318 (PMC5904206; doi:10.3389/fphar.2018.00318)
Supplement: Supplementary file 1 [file Table_1.pdf]

Supplemental Table 1

Echocardiographic indices of WT, untreated TazKD and BF-treated TazKD mice at 3, 5 and 7 months of age.

Group	WT			TazKD			TazKD + BF		
Age, months	3	5	7	3	5	7	3	5	7
IVS;d	0.81±0.15	0.84±0.08	0.91±0.17	0.95±0.11*	1.04±0.18#	0.95±0.10	0.89±0.14	1.04±0.16	0.87±0.11
IVS;s	1.44±0.26	1.27±0.13	1.34±0.24	1.49±0.16	1.47±0.27	1.34±0.14	1.46±0.17	1.50±0.24	1.29±0.14
LVID;d	3.97±0.38	3.91±0.46	3.92±0.48	4.02±0.50	4.16±0.50	4.19±0.52	3.80±0.17	3.48±0.25*	3.63±0.32*
LVID;s	2.50±0.48	2.76±0.51	2.67±0.56	2.56±0.33	2.82±0.75	3.21±0.55#	2.35±0.18	2.32±0.39	2.43±0.21*
LVPW;d	0.79±0.12	0.77±0.10	0.86±0.10	0.93±0.11#	0.84±0.12	0.87±0.15	0.84±0.12	0.89±0.11	0.79±0.14
LVPW;s	1.22±0.18	1.16±0.18	1.31±0.20	1.56±0.41#	1.19±0.19	1.13±0.16#	1.32±0.15	1.33±0.27	1.16±0.12
EF	67.16±10.25	57.07±9.79	59.68±11.72	65.01±5.33	52.82±12.96	47.82±7.03#	65.01±8.41	49.66±6.53	60.88±6.05*
FS	37.27±7.51	29.83±6.69	30.16±7.85	36.07±5.42	27.11±8.49	23.75±4.66#	36.07±5.42	25.37±3.90	32.56±9.29*
LV Mass	92.72±14.87	91.41±17.87	103.37±19.42	95.81±14.82	114.90±29.09#	115.76±28.17	91.88±23.27	103.86±14.50	84.37±14.11*
LV Vol;d	69.69±15.55	67.76±19.67	67.70±20.61	64.60±4.94	78.18±21.77	84.28±13.14#	60.39±5.86	51.86±9.50*	55.78±11.38*
LV Vol;s	23.59±12.40	30.00±14.06	28.42±14.52	22.54±3.45	38.42±18.17	44.22±10.19#	20.86±4.96	26.07±5.94*	21.66±4.40*
HR	438±90	414±67	462±91	353±59#	395±71	404±52	339±73	393±66	410±68

Data are presented as means ± standard deviation.
IVS – diastolic (d) and systolic (s) interventricular septal wall thickness (mm);
LVID – diastolic (d) and systolic (s) left ventricular internal dimension (mm).
LVPW – diastolic (d) and systolic (s) left ventricular posterior wall thickness (mm).
EF – left ventricular ejection fraction (%).
FS – left ventricular fractional shortening (%).
LV Vol – diastolic (d) and systolic (s) left ventricular diastolic volume (microliters).
HR, heart rate (beats/min).
Statistically significant differences ($p < 0.05$) are indicated: *, between untreated and BF-treated TazKD mice of same age group; #, between untreated WT and TazKD mice of same age group.
