# Supplementary material for: The Effects of PPAR Stimulation on Cardiac Metabolic Pathways in Barth Syndrome Mice
Source: Front Pharmacol. 2018 Apr 11;9:318. doi: 10.3389/fphar.2018.00318 (PMC5904206; doi:10.3389/fphar.2018.00318)
Supplement: Supplementary file 5 [file Image_1.pdf]

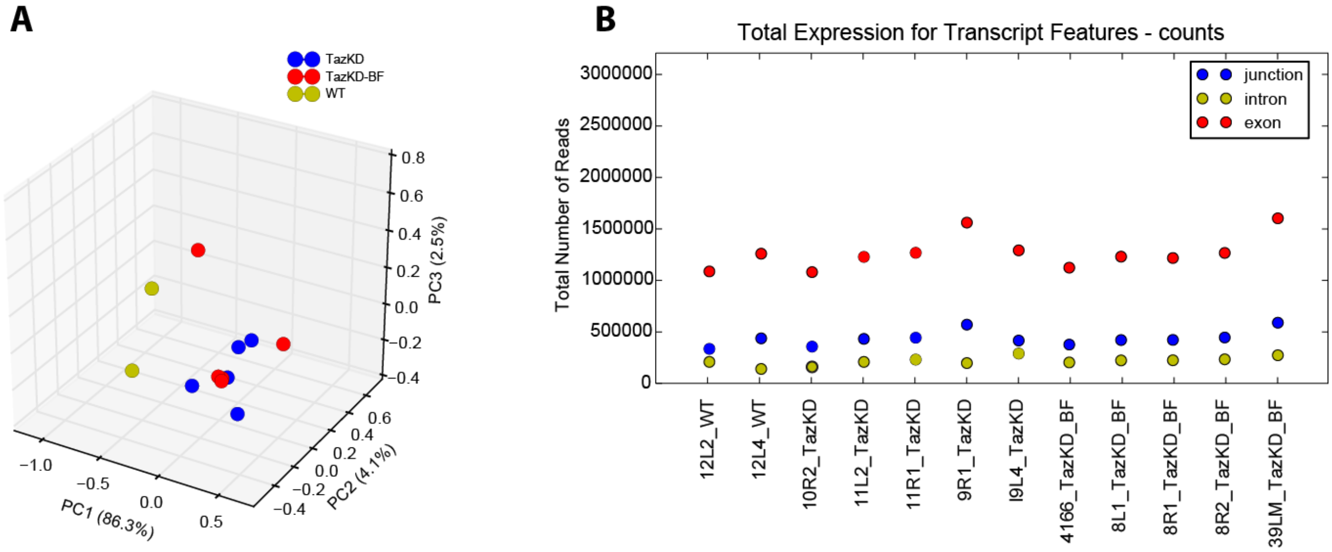

Supplemental Figure 1. A – Dimensionality reduction with principal component analysis wit Z-score normalization. B – Counts of total transcript features for all individual samples submitted for RNA-seq.
