# Supplementary material for: The Effects of PPAR Stimulation on Cardiac Metabolic Pathways in Barth Syndrome Mice
Source: Front Pharmacol. 2018 Apr 11;9:318. doi: 10.3389/fphar.2018.00318 (PMC5904206; doi:10.3389/fphar.2018.00318)
Supplement: Supplementary file 6 [file Image_2.pdf]

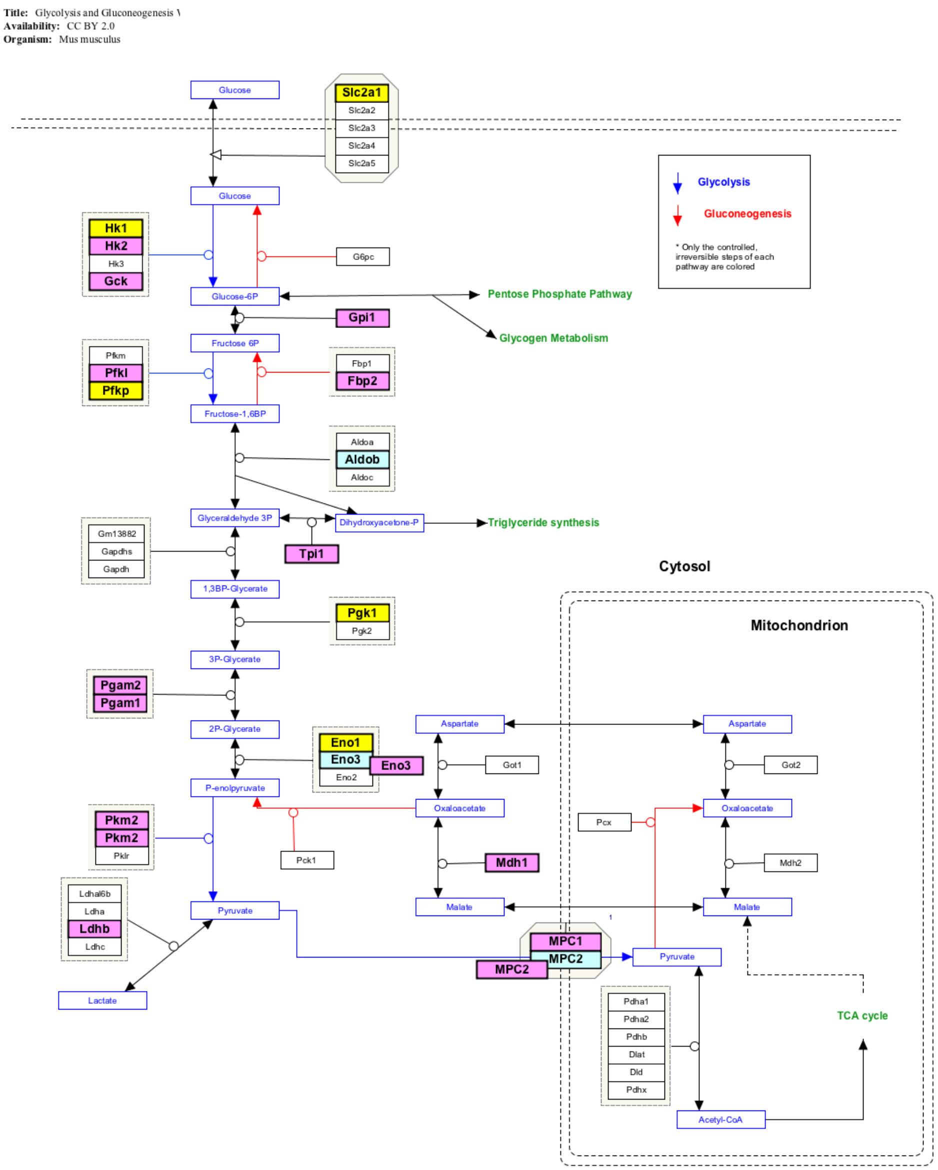

Supplemental Figure 2. Glycolysis and gluconeogenesis pathway (WP157). For supplementary figures 2-11: a yellow color highlights the genes, which are upregulated in TazKD group compared to WT controls; a purple color highlights the genes, which are upregulated in BF-treated TazKD group compared to untreated TazKD group; blue highlights the genes, which are downregulated in untreated TazKD group, but upregulated in BF-treated TazKD group. Pathways are derived from WikiPathways (http://www.wikipathways.org).
