# Supplementary figures and images for: The Effects of PPAR Stimulation on Cardiac Metabolic Pathways in Barth Syndrome Mice
Source: Front Pharmacol. 2018 Apr 11;9:318. doi: 10.3389/fphar.2018.00318 (PMC5904206; doi:10.3389/fphar.2018.00318)

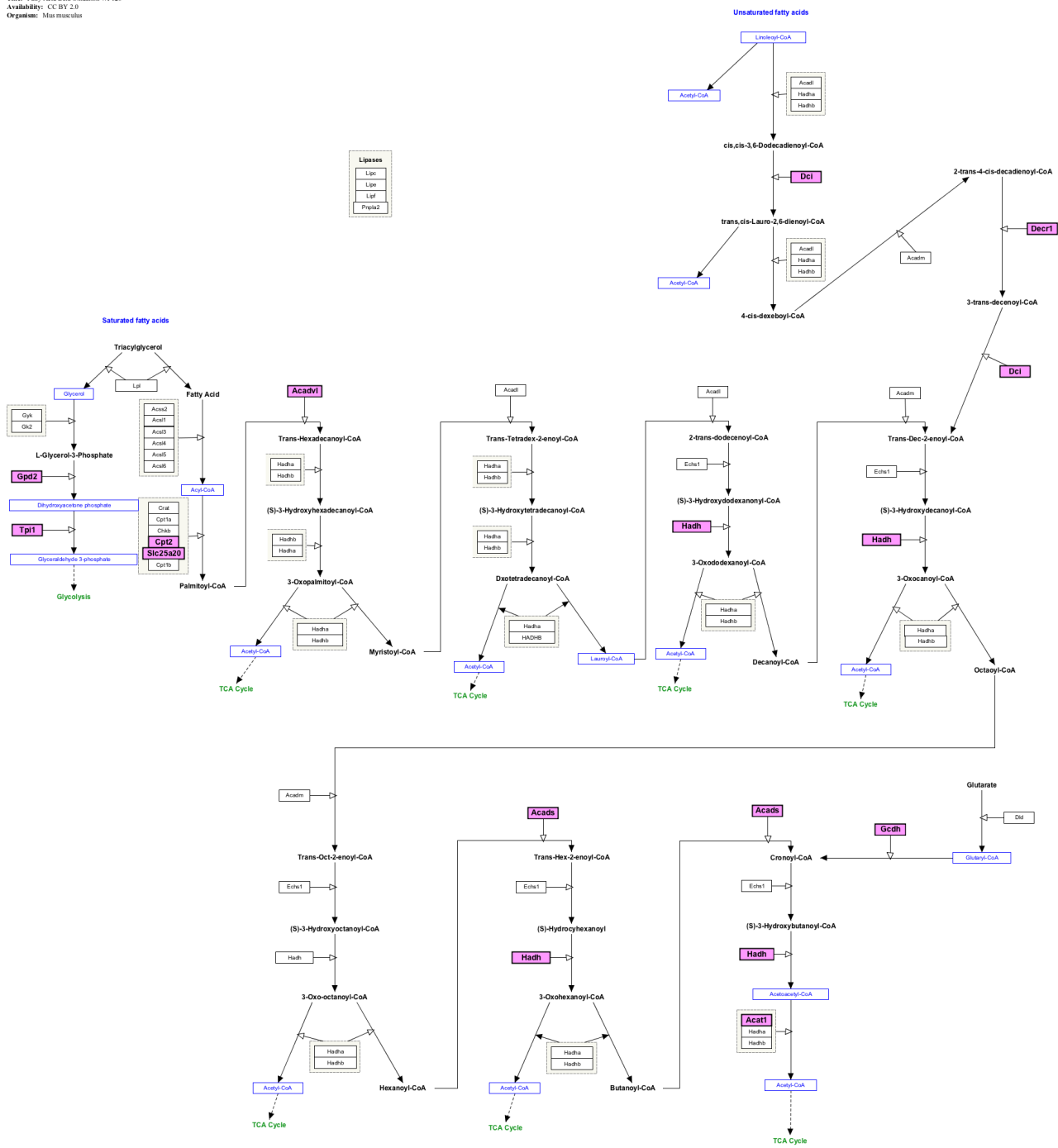

**Supplemental Figure 3.** Fatty acid beta-oxidation pathway (WP1269).

Supplement: Supplementary file 7 [file Image_3.pdf]

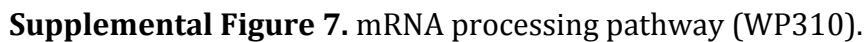

Supplement: Supplementary file 11 [file Image_7.pdf]

Title: G13 Signaling Pathway  
Organism: Mus musculus

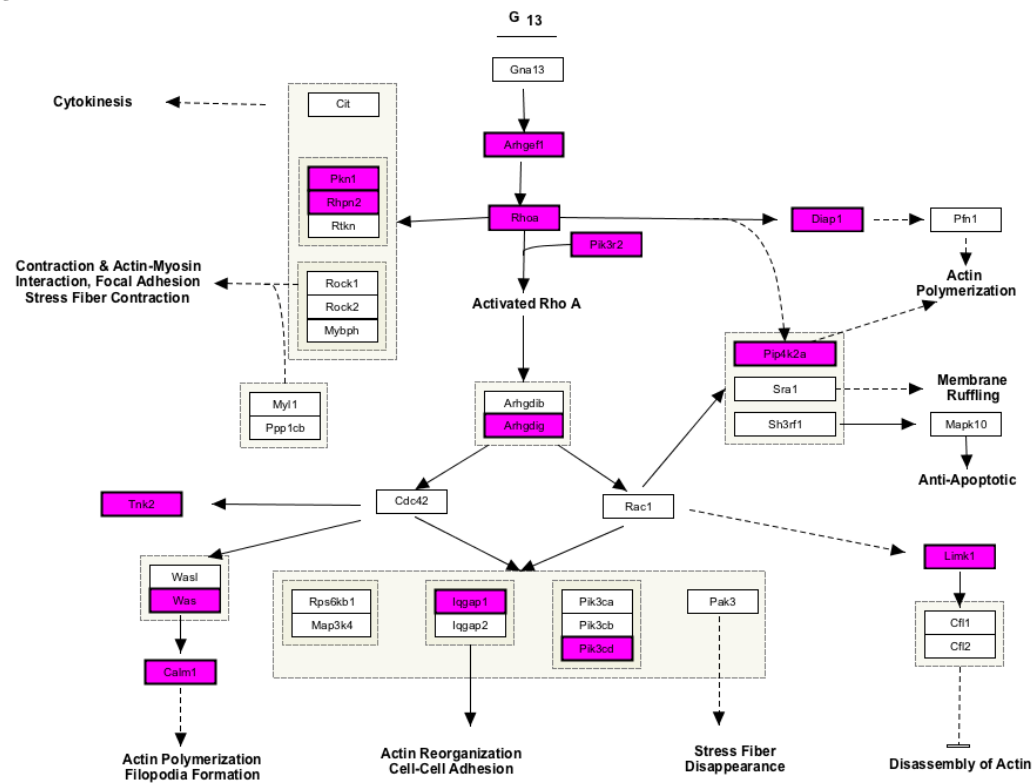

Supplemental Figure 9. G13 signaling pathway (WP298).

Supplement: Supplementary file 13 [file Image_9.pdf]
