# Supplementary material for: The Effects of PPAR Stimulation on Cardiac Metabolic Pathways in Barth Syndrome Mice
Source: Front Pharmacol. 2018 Apr 11;9:318. doi: 10.3389/fphar.2018.00318 (PMC5904206; doi:10.3389/fphar.2018.00318)
Supplement: Supplementary file 8 [file Image_4.pdf]

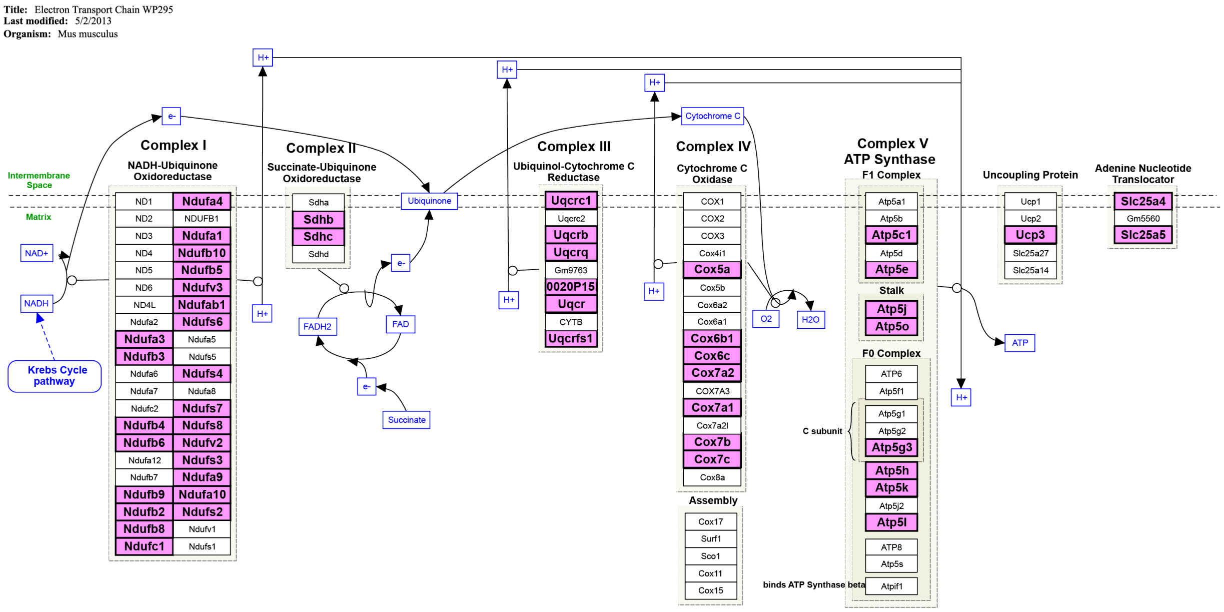

Supplemental Figure 4. Oxidative phosphorylation (WP1248) and electron transport chain (WP295).
