# Supplementary material for: The Effects of PPAR Stimulation on Cardiac Metabolic Pathways in Barth Syndrome Mice
Source: Front Pharmacol. 2018 Apr 11;9:318. doi: 10.3389/fphar.2018.00318 (PMC5904206; doi:10.3389/fphar.2018.00318)
Supplement: Supplementary file 15 [file Image_11.pdf]

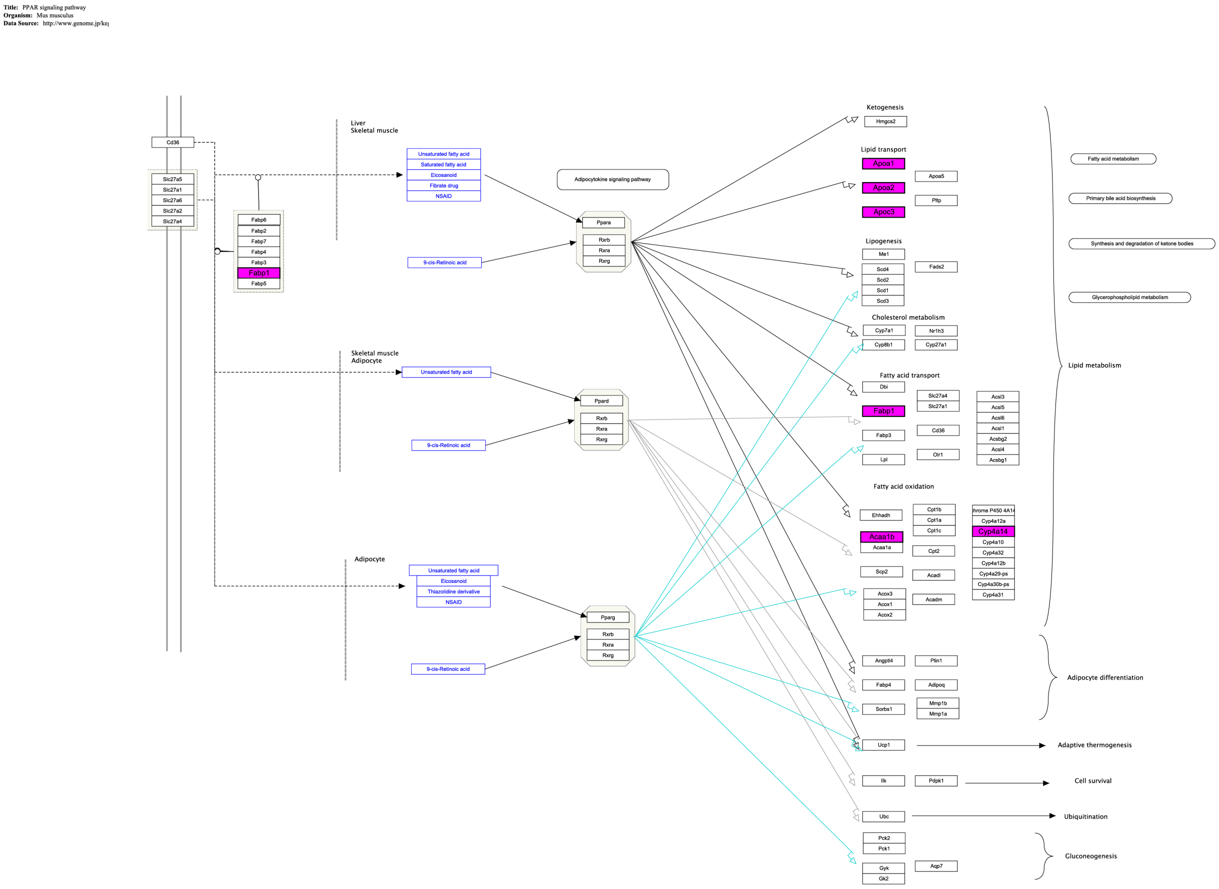

Supplemental Figure 11. PPAR signaling pathway (WP2316).
